# A robotic system for researching social integration in honeybees

**DOI:** 10.1371/journal.pone.0181977

**Published:** 2017-08-09

**Authors:** Karlo Griparić, Tomislav Haus, Damjan Miklić, Marsela Polić, Stjepan Bogdan

**Affiliations:** University of Zagreb, Faculty of Electrical Engineering and Computing, Zagreb, Croatia; Tongji University, CHINA

## Abstract

In this paper, we present a novel robotic system developed for researching collective social mechanisms in a biohybrid society of robots and honeybees. The potential for distributed coordination, as observed in nature in many different animal species, has caused an increased interest in collective behaviour research in recent years because of its applicability to a broad spectrum of technical systems requiring robust multi-agent control. One of the main problems is understanding the mechanisms driving the emergence of collective behaviour of social animals. With the aim of deepening the knowledge in this field, we have designed a multi-robot system capable of interacting with honeybees within an experimental arena. The final product, stationary autonomous robot units, designed by specificaly considering the physical, sensorimotor and behavioral characteristics of the honeybees (*lat. Apis mallifera*), are equipped with sensing, actuating, computation, and communication capabilities that enable the measurement of relevant environmental states, such as honeybee presence, and adequate response to the measurements by generating heat, vibration and airflow. The coordination among robots in the developed system is established using distributed controllers. The cooperation between the two different types of collective systems is realized by means of a consensus algorithm, enabling the honeybees and the robots to achieve a common objective. Presented results, obtained within ASSISIbf project, show successful cooperation indicating its potential for future applications.

## Introduction

Cooperation among individuals inside a group of social animals can induce a complex behaviour of the group, enabling it to handle tasks not manageable by an individual alone [1]. This robust and adaptive group behaviour is responsible for efficient performing of everyday activities of animals, such as foraging, reproducing and surviving. The motivation for understanding the principles of collective behaviour in social animals stems from the possibility of using the existing, already optimized evolutionary mechanisms of decision making, and applying them in developing technical systems requiring robust, decentralized and efficient functionality, through the concept of biomimicry [2]. From a broader point of view, the principles of collective behaviour can explain various phenomena in social sciences (actions taken by a large group of people in case of emergency [3]), economics (heavy tails in the distribution of returns [4]), physics (ordered state with collective global properties as a consequence of a system of individual particles that interact locally [5]), just to mention few. Among other complex natural phenomena, collective behaviour is especially interesting for researchers because of its apparent universality, which enables its deployment in different systems that only resemble the systems where it was first observed under loose conditions. Here, local interaction and partial information of individuals, along with the decision algorithm, play the most important role. Since these conditions are easily satisfied, the evolution of this mechanism in different environments does not surprise, with examples of collective behaviour found in flocks of birds, schools of fish, insect colonies, and other groups. It is clear even at a glance that this mechanism does not depend on the group size or other physical properties, yet always guides a group of individuals autonomously towards a common objective. Therefore, the focus is mainly on describing the individual agents and their relations, rather than describing the complex behaviour of a group as a whole.

Mathematical models based on behavioural algorithms have become a tool for describing complex natural systems [6], with a possible application of such models to the control of distributed technical systems as a prospect. However, the key challenge lies in developing adequate methods for extracting collective behaviour principles from natural societies. According to [7], behaviour of the individuals inside a group of social animals depends on their interaction with others in the group. Robots designed in such a way that they can generate particular stimuli and interact with animals, which consequently leads to acceptance of robots by animals as a part of their society, have shown a huge potential in animal behaviour research.

It is clear that stimuli that can be accepted by particular animal play essential role in interaction. For example, interactive abilities of robots that bear resemblance to an animal are presented in [8, 9] where fish replica was used. Based on investigation of fish movement, the authors were able to plan motion trajectories of the mobile robot (situated below the experimental setup) driving fish replica, so that the replica was accepted by animals. Additional step towards increased acceptance, described in [10, 11], was development of a moving tail that can mimic body movements of a fish. Another example of robotic replica able to actively generate stimuli is given in the study of social behaviour of the bowerbird [12], where a robot was used for male courtship response.

Due to very small dimension of honeybees, for the research presented herein more significant were results obtained by robots that are quite different in size and shape of animals they interact with. Wheeled miniature mobile robots treated with attractive chemical clue were used in ethological experiments with cockroaches [13, 14]. It has been shown that animals accepted robots as their equals, so that robots became members of the animal society. Another example is a mobile robot able to generate visual and acoustic stimuli that was introduced for social integration in a group of chickens [15]. An important insight that these experiments clearly yielded is the feasibility of interaction with animal society by generating attractive or repellent stimulation, with a robot that does not necessarily physically resemble the animal it is interacting with. This fact was of the prime importance when we considered design of our system.

Among many scientifically interesting species, the honeybees have been chosen for collective intelligence exploration not only because of their important impact on human society, but also because of many interesting phenomena that emerge within a group due to intricate communication queues and complex honeybee society structure. Different aspects of their subtle behaviour have been the subject of intensive research in biology for decades, including the waggle dance, which can exhibit a range of signals for food location and availability [16], thermotactic aggregation [17], and vibration freezing effect [18]. To explore models and algorithms responsible for collective decision making in the honeybee society, in contrast with [19] where a honeybee size robot was developed whose motion is controlled in two-dimensional space for the study of honeybee dance communication system, we developed a network of novel interactive robots equipped with relevant actuating and sensing capabilities. The network is comprised of stationary robots, called CASUs (Combined Actuator-Sensor Units), equally distributed in an experimental arena where non flying juvenile honeybees can move freely.

Having in mind limited sensing and operating range of previously used interactive robots, as well as the drawbacks of their centralized visual feedback approach that neglected information of other physical states, and taking into consideration the nature of a biological system used in presented research (a group of social animals) a multi-robot approach self-imposed as a logical technique of choice for exploration and integration into natural society. If one considers the number of interactions between individuals (in our case honeybees) within a group per time unit, then the only way to achieve similar order of interactions between animals and technical system is usage of multiple robots. Currently, due to limited computational power, as well as sensors and actuators dimensions, interactive capabilities of a single robot are far below the level required for successful cooperation with large animal groups. The multi-robot approach overcomes these computational and physical constraints through distribution. The concept of a network of stationary units was chosen, instead of designing mobile robots, because of its advantages regarding constraints on size, stimuli generation and energy demand. This concept has been introduced in [20], where the reaction of honeybees on physical stimuli was experimentally evaluated.

The increased robustness of multi-robot systems, due to both decentralization and redundancy, extends the field of possible applications making the system adaptive to environmental conditions and less constrained by physical limitations. Additionally, the flexibility of such systems enables them to solve problems more efficiently in a distributed manner through parallelism, again in a wide variety of applications and robustly through reconfigurability and adaptation. With a set of capabilities and characteristics as described, multi-robot systems have been proven successful in solving problems impossible for single robot operation [21]. Finally, stemming mainly from decentralization and redundancy, the feasibility of such systems is increased by energy and electro-mechanical cost efficiency because of exclusion of expensive centralised sensory, actuator, and computation units upon which single robot systems usually depend. These advantages of multi-robot systems justify their deployment even in applications solvable by single robots, but in a faster, more efficient, more robust, or cheaper way.

Where a decentralized approach is chosen, multiple physical constraints such as communication ranges, energy distribution and other limitations can be overcome, however at the expense of even higher and more complex structure and organization of control [22]. Along with multi-robot systems development, adequate control and coordination mechanisms are being developed simultaneously. A set of independent, relatively simple and limited units needs to be guided by an intelligent algorithm in order to fully achieve its potential, beyond the sole inherent redundancy. In a pursuit of such an algorithm, there are several requirements that need to be met. First of all, it is necessary to maintain a locally based consistent view of the common objective and current state of the system among all of the individuals. Additionally, this kind of cooperation is to be conducted based on information that can only be propagated and updated through the network of robots by means of local neighbour to neighbour interaction. One of the prominent distributed control algorithms that satisfies these requirements, with many more additional desirable properties, such as time-delay robustness, is the consensus algorithm [23–25], which is therefore also deployed in this work.

This paper describes the developed system starting from the requirements that needed to be met in order to achieve robot integration with a group of juvenile honeybees. Mechanical and electrical properties of the designed system are described in detail, with emphasis on the modularity, scalability, and hardware architecture of a decentralized multi-robot system. The control algorithm is explained along with the mathematical assumptions and model of the desired system dynamics. Verification of the system functionality has been conducted by a set of experiments planned so that i) local interactions between CASUs and honeybees were confirmed, and ii) collective decision was done in decentralized manner. Local interactions have been tested in both directions—honeybees influence CASUs and CASUs influence honeybees. Namely, during experiments, each CASU, by using its IR proximity sensors, measured local density (presence) of honeybees and communicated measurements to its neighbors. By using decentralized consensus algorithm, CASUs agreed on the position of the CASU with the highest honeybees density. This in turn caused formation of the temperature field stimulus so that CASUs influenced honeybees and they aggregated in vicinity of CASU with the highest density. Presented experimental results demonstrate, for the first time, successful interaction between honeybee groups and an interactive multi-robot system.

## Materials and methods

### Animals

In the experiments presented in this paper we use young honeybees (*lat. Apis mellifera*) aged from 1 to 30 hours. Their maximum body length, height and width are 10 mm, 5 mm and 3 mm, respectively. These bees cannot fly and cannot produce heat. However, it has been shown that they are extremely sensitive to heat and prefer temperature of 36°*C* [26]. Based on these results, heat has been chosen in our setup as an attractive stimulus for honeybees. Furthermore, vibration patterns have been shown to cause a “freeze” effect on honeybees [18]. Therefore, vibrations have been chosen as a stopping stimulus. Finally, we complete our stimuli list with airflow as a repellent stimulus. To the best of our knowledge, this stimulus has not been yet used in experiments with honeybees, but during the experiments conducted within ASSISIbf project in the last four years, it has been observed that honeybees avoid areas with active airflow.

Before the experiments, the honeybees are kept in a plastic box and fed honey. Each bee is used only in a single experiment and it is placed in another plastic box after the experiment. At the end of the day, all bees are returned to their original hive.

### Experimental setup

**Fig 1:**
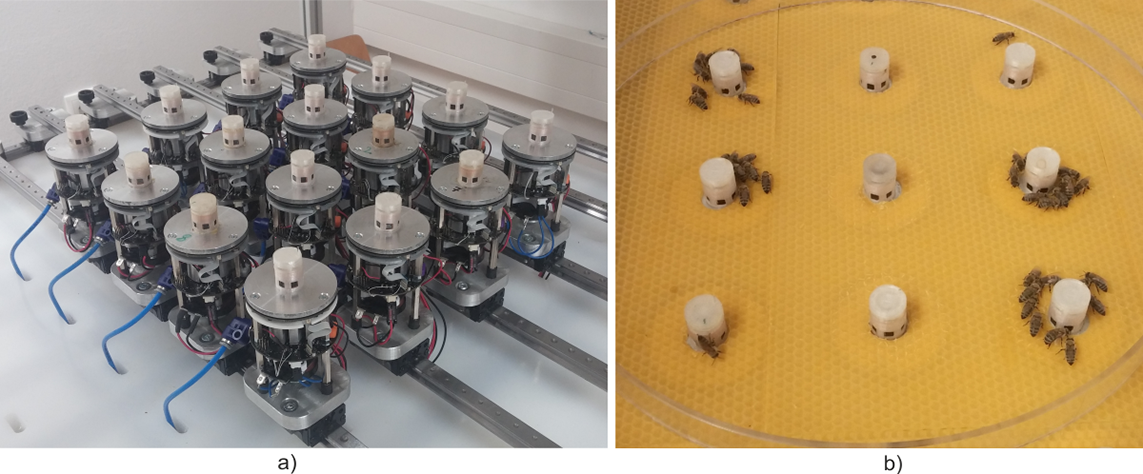
The developed experimental setup. (a) A network of 16 static CASUs without arena floor. (b) The interactive arena with honeybees.

Since the aim of the experimental arena is to provide enough possibilities for honeybees to choose their preferred behaviour or state, we developed a network of cooperating CASUs situated in a circular arena with a maximum diameter of 850 mm. The CASU has been designed as a modular robot, with variable number and flexible position inside the arena. The experimental arena is housed within a plastic frame that integrates the external infrastructure needed for operation of the designed static multi-robot network, including a positioning system, Ethernet network infrastructure, power supplies and compressed air system. The developed experimental setup is equipped with a guide rails mechanism, designed to meet scalability and modularity requirements of the described static multi-robot system, as presented in Fig 1. The railing mechanism enables variable CASU placement in two-dimensional space necessary for different network topology realizations.

Comprehensive description of the design of the experimental setup and CASU can been found in S1 Technical Documentation.

#### CASU design

The main purpose of the CASU mechanical construction is to effectively transfer selected stimuli to the ambient. Besides actuators, the mechanical construction houses the sensory units for temperature and vibration measurements, as well as honeybee presence detection. From the honeybee perspective, the CASU is separated by the floor into two parts, visible and non-visible.

The visible part is designed with the requirement that unit size should be comparable with size of a honeybee. This plastic circular body, with a diameter of 18.5 mm, contains a diagnostic light, six proximity sensors, a temperature sensor, and nozzles for airflow. The programmable diagnostic light visible at the top of the CASU is designed considering the need for user evaluation of the CASU operation. Proximity sensors situated inside the CASU body are used for honeybee presence detection measurement.

**Fig 2:**
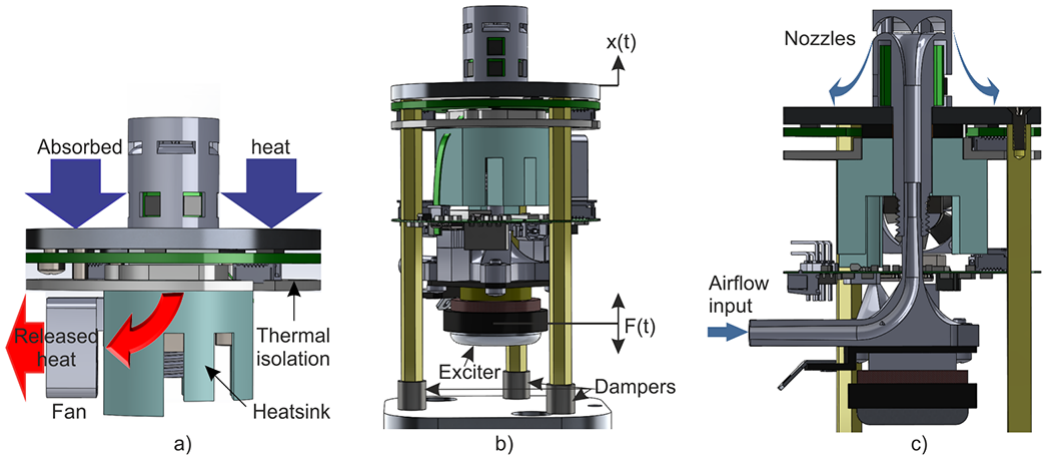
Operation principle of CASU stimuli. (a) In the cooling mode, the temperature of the ring decreases, resulting in the absorption of heat from the ambient. The other side of the Peltier module releases heat through the cooling system. (b) Oscillatory displacement of the CASU is generated by external force. (c) Cross section shows the air pipe inside the CASU.

The non-visible part of a CASU is covered by wax of the arena floor during experiments. Directly under the top aluminum ring, the CASU body houses components for thermal control of the arena floor (Fig 2a), of which an active element responsible for thermal conduction is the thermoelectric Peltier module. Further below, other components for stimuli production are housed. The freezing effect can be triggered using an exciter mounted on the bottom of the CASU. The inertia of the exciters moving mass produces the oscillatory response of the whole CASU body in form of mechanical vibrations, which results in vertical displacement of arena floor in close surroundings of the CASU (Fig 2b). The repelling stimulus is enabled by a built in airflow channel conducted from the remote air compressor pump through the CASU body (Fig 2c). Additionally, all of the computation power and assistive components are hidden within this non-visible part.

Since honeybee reaction can easily be influenced by any heat source, actuators and electronics thermal dissipation must be isolated from the arena floor. All of the electronic components are therefore located on the bottom side of the heatsink in the proposed solution. This design decision additionally guarantees temperature stability of the ring in the presence of external thermal disturbances while the system is under closed loop temperature control.

**Fig 3:**
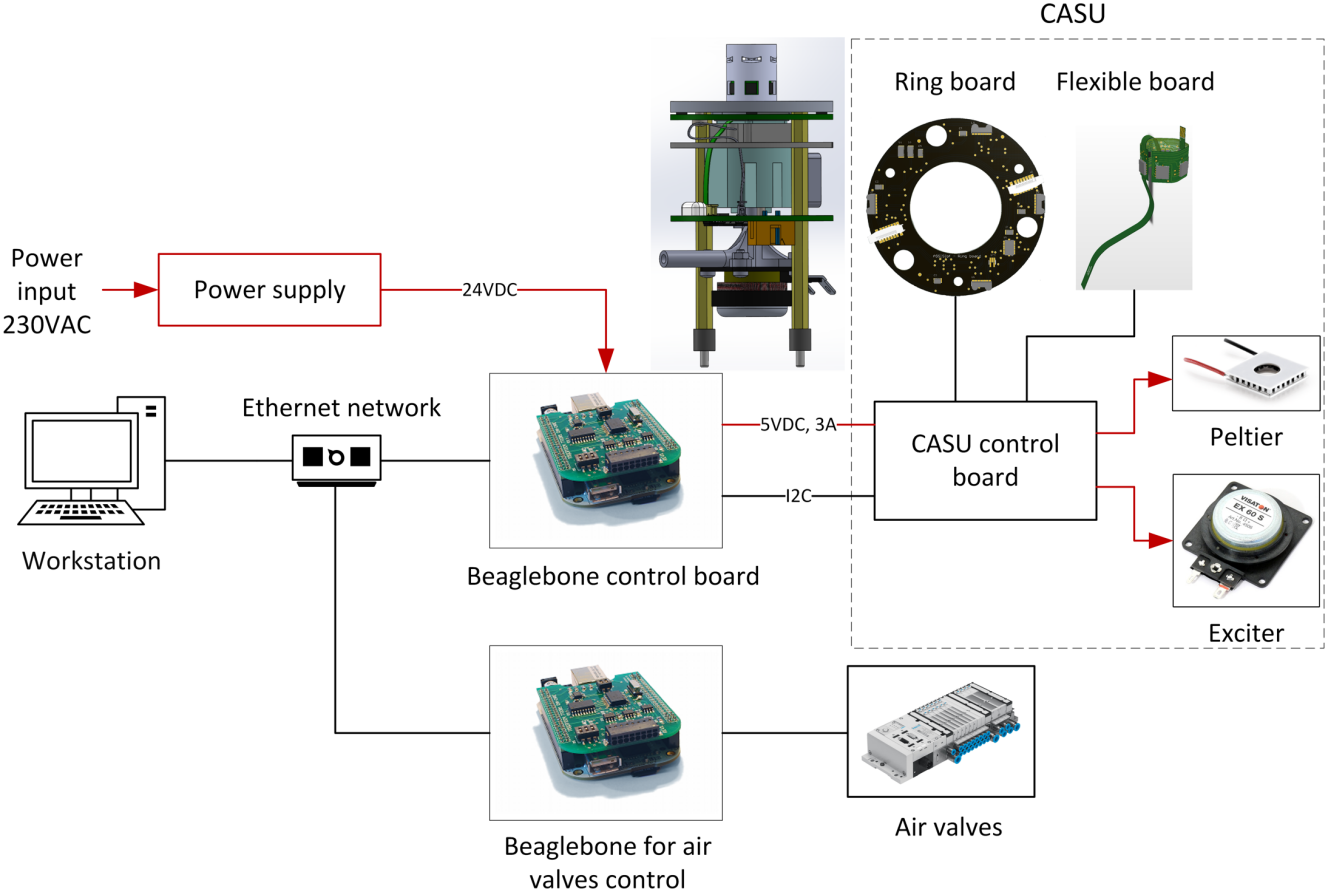
Electronic architecture diagram. The control architecture is hierarchically divided into two levels, where black lines show signal connections between components and red arcs show energy flow. The low-level stage includes electronic components controlled by two microcontrollers. *Beaglebone* computer, on which user control algorithms are executed, represents the high-level stage.

The designed electronic architecture, hierarchically divided into two levels, integrates peripheral capabilities for connecting actuators and sensors, and processing power for control program execution, in order to integrate interactive requirements and develop a decentralized network of CASUs. The electronic architecture diagram is shown in Fig 3.

The low-level architecture is responsible for the management of sensors and actuators, i.e., reading raw sensor data and controlling actuator outputs. The high-level side of the electronic architecture, based on a *Beaglebone* computer, provides an interface between the high-level software and the low-level CASU functionalities through the developed Python API (Application Programming Interface). The computer independently executes each user program and communicates with other CASU user programs on the network.

Each CASU has several common components including power supply, local Ethernet network infrastructure, air valves with control board, and an operator workstation that provides programming and monitoring functionalities for the designed multi-robot system.

### Algorithms for honeybee interaction

In [26], the collective decision making process of juvenile honeybees exposed to a static thermal environment has been investigated. Inspired by the presented self-organising behaviour of honeybees, we designed experiments with the aim of achieving the aggregation of honeybees inside a two-dimensional arena using the designed robotic system, where aggregation is quantified as the percentage of the honeybees located around a certain CASU. The network of CASUs uses heat stimulation to create an actively changing complex thermal environment that is directly influenced by honeybee distribution in the arena. At the same time the thermal gradient created by CASUs drives honeybees towards the warmest point in the arena. According to the experiment scenario, the CASU with the highest honeybee density measurement becomes a leader, and sets its temperature reference value to 36°*C*. The other CASUs in the network calculate their temperature references depending on the highest temperature of their neighbours. Coordination between the CASUs is implemented in a decentralized manner, using consensus algorithm for density measurement propagation of each CASU.

**Fig 4:**
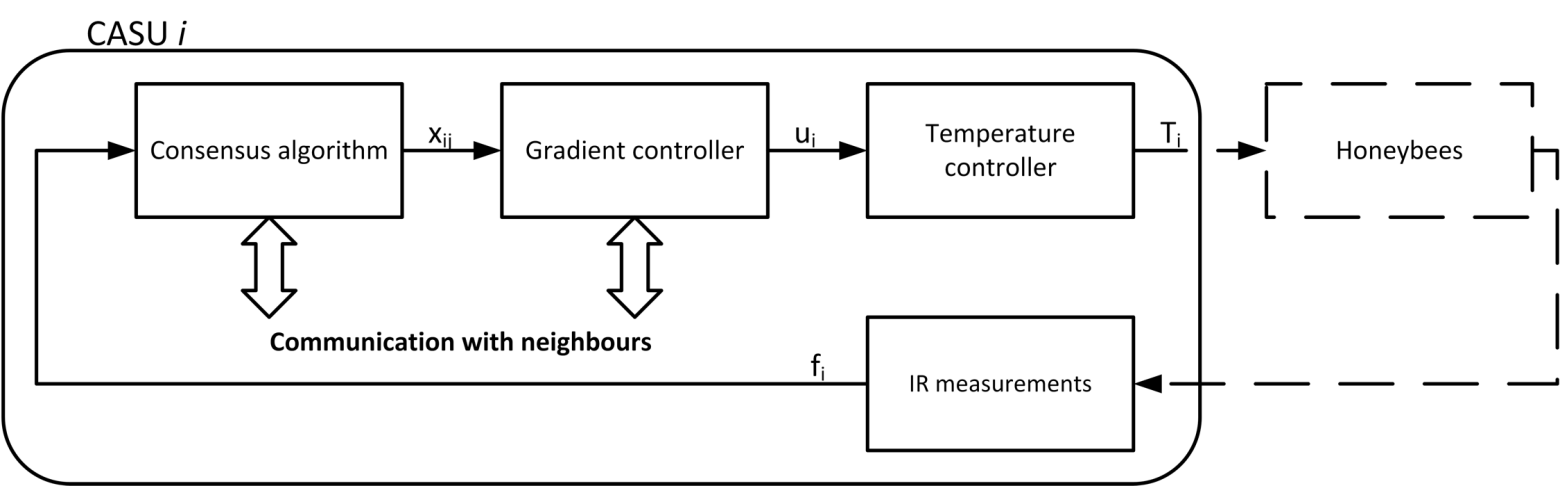
The control architecture of an individual CASU. The block diagram shows that the biological society influences a CASU through its density measurement. The consensus algorithm propagates density estimates among all CASUs in the network, and based on its outputs the gradient controller calculates temperature setpoints for a low-level control law to achieve the desired temperature.

The CASU control architecture block diagram is presented in Fig 4. The density measurement of a CASU is obtained from the proximity sensor activity. A sensor is considered to have detected an object, i.e. is activated, if its measurement surpasses its threshold, obtained through calibration at the beginning of each experiment. Depending on the presence and movement of honeybees in the close surroundings of a CASU at each time step of the experiment, the activity of the proximity sensors varies, and the density estimates, defined by the total number of activated proximity sensors, is calculated for each CASU. Each CASU computes its local vector of density values *x*_*ij*_ of all other CASUs in the network based on consensus algorithm that propagates measurements by exchanging data among neighbours. The gradient controller then determines the temperature reference for each CASU, according to its respective density vector and the reference temperatures of its neighbours. The CASU heat controller refers to the low-level ring temperature control loop implemented using PI controller.

#### Consensus algorithm

We introduce graph theory notations and basic concepts necessary for understanding our implementation of the consensus algorithm. A graph G=(V,E) consists of a set of vertices V={1,2,…,N}, where *N* is the total number of vertices in the graph, and a set of edges E⊂V×V representing connections between vertices. A graph can be directed or undirected depending on the assigned direction on its edges. The neighbours that are adjacent to vertex *i* are called the neighbours of *i* and this set is denoted as $N_{i}=\left\{v_{j}\in V:e_{ij}\in E\right\}$. There are various matrices that can be associated with a graph and reflect its properties. In this paper we use the adjacency matrix $A\in R^{N\times N}$, which defines the connection of the vertices in the graph. If vertex *i* is adjacent with vertex *j*, then adjacency matrix element *a*_*ij*_ is equal to 1, otherwise *a*_*ij*_ = 0. The adjacency matrix is symmetric *a*_*ij*_ = *a*_*ji*_ for an undirected graph. If a graph *G* is weighted, then adjacency elements $a_{ij}\geq 0,\in R$ if $e_{ij}\in E$.

Consider a network of *n* agents, where each agent’s dynamics can be described with the following equation:

$$\begin{array}{c} {\dot{x}}_{i}\left(t\right)=r_{i}\left(t\right),\;i\in \left\{1,2,\ldots ,n\right\}. \end{array}$$ (1)

where $x_{i}\left(t\right)\in R$ is the state and $r_{i}\left(t\right)\in R$ is the control input of the *i*th agent. The agent’s state update using the consensus algorithm is given by:

$$\begin{array}{c} {\dot{x}}_{i}\left(t\right)=\sum_{j\in N_{i}}a_{ij}\left(x_{j}\left(t\right)-x_{i}\left(t\right)\right), \end{array}$$ (2)

where *a*_*ij*_ is the adjacency matrix element and $N_{i}$ is a set of neighbours of agent *i*. With the assumption that the graph G is undirected and connected, Eq (2) globally asymptotically converges for all initial states [27].

As described in the previous section, an agent becomes a leader if its measured honeybee density is the highest among other agents. Thus, each agent should compare its own measurement with measurements of all agents in the network. Since each agent can communicate only with its neighbours, the density value of each agent should be propagated through the network. In other words, each agent should have its own state vector that contains density measurements of all agents. We modified the trust based consensus algorithm presented in [28], where we implemented the observation function using density measurement of honeybees, and proposed a new way for calculating of the weight of communication links between neighbouring agents. We are proposing the following algorithm for the measurement vector update of agent *i*:

$$\begin{array}{c} {\dot{x}}_{ij}\left(t\right)=\{\begin{array}{cc} \sum_{l\in N_{i}}a_{il}\left(x_{lj}\left(t\right)-x_{ij}\left(t\right)\right)+\left(f_{i}\left(t\right)-x_{ij}\left(t\right)\right), & i=j,\;(3\text{a})\\ \sum_{l\in N_{i}}a_{il}\left(x_{lj}\left(t\right)-x_{ij}\left(t\right)\right), & i\neq j,\;(3\text{b}) \end{array} \end{array}$$

where *a*_*il*_ is the (*i*, *l*) element of the adjacency matrix associated to the network graph, *x*_*ij*_ is agent’s *j* density measurement seen by agent *i*, and *f*_*i*_(*t*) is the density function of agent *i*.

Since the algorithm is commonly executed at discrete time steps *k* ∈ {0, 1, 2, …}, it can be represented in a discrete form:

$$\begin{array}{c} x_{ij}\left(k+1\right)=x_{ij}\left(k\right)+\epsilon \cdot \Delta x_{ij}\left(k\right) \end{array}$$ (4)

$$\begin{array}{c} \Delta x_{ij}\left(k\right)=\{\begin{array}{cc} \sum_{l\in N_{i}}a_{il}\left(k\right)\left(x_{lj}\left(k\right)-x_{ij}\left(k\right)\right)+\left(f_{i}\left(k\right)-x_{ij}\left(k\right)\right), & i=j,\;(5\text{a})\\ \sum_{l\in N_{i}}a_{il}\left(k\right)\left(x_{lj}\left(k\right)-x_{ij}\left(k\right)\right), & i\neq j,\;(5\text{b}) \end{array} \end{array}$$

where *ϵ* > 0 is the agent’s sample time, agent *i* density function $f_{raw}^{i}\left(k\right)$ is defined as a sum of activated proximity sensors *d*_*n*_(*k*) divided by the total number of CASU’s sensors in each time step *k*:

$$\begin{array}{c} f_{raw}^{i}\left(k\right)=\frac{\sum_{n=1}^{6}d_{n}\left(k\right)}{6}. \end{array}$$ (6)

The density function *f*_*i*_(*k*) is additionally smoothed with a moving average filter with a window of size *M* = 5, given by:

$$\begin{array}{c} f_{i}\left(k\right)=\frac{\sum_{m=0}^{M-1}f_{raw}^{i}\left(k-m\right)}{M}. \end{array}$$ (7)

Based on the presented algorithm (Eq (5)), it should be noted that the execution of the algorithm on each agent is conducted in a completely asynchronous way. A new value of the state vector is calculated based on the current measured values and received data from agent’s neighbours. We defined the adjacency matrix element of agent *i* *a*_*il*_(*k*) ∈ [0, 1] as the ratio of the received number of messages *N*_*l*_ from agent *l* and maximal number of messages *N*_*max*_ that can be exchanged between neighbouring agents determined by the throughput of the used communication channel:

$$\begin{array}{c} a_{il}\left(k\right)=\frac{N_{l}}{N_{max}}. \end{array}$$ (8)

Therefore, the topology of the multi-robot system can be described with a dynamical graph G=(V,E(k)), where elements of the adjacency matrix change over time. Since the topology is changing, it should be noted that the convergence speed of the consensus is influenced by the number of topological neighbours, as has been proven in [29]. Additionally, using a hybrid metric-topological distance role for creation of connections between neighbouring agents, where the agent’s set of neighbours is correlated with the number and distance between agents, as presented in [30], the limitations of agent’s sensing and computational abilities are overcome. However, even though the topology of the multi-robot system plays an crucial role on the performance of the consensus algorithm, the connectivity maintenance of the network is beyond the scope of this paper, and we assume that each agent is connected with its nearest neighbours and that the graph is connected for every time step *k*.

#### Gradient controller

The gradient controller calculates the temperature setpoint *u*_*i*_ based on the measurement vector of CASU *i*. A set of maximum values of the agent’s density vector *x*_*ij*_(*k*) is defined as:

$$\begin{array}{c} M_{i}\left(k\right)=\max_{j}x_{ij}\left(k\right). \end{array}$$ (9)

The temperature setpoint of agent *i* is updated using the following equation:

$$\begin{array}{c} u_{i}\left(k+1\right)=\{\begin{array}{cc} T_{leader}, & x_{ii}\in M_{i}\left(k\right):\left|M_{i}\left(k\right)\right|=1,\;\left(10\text{a}\right)\\ u_{i}\left(k\right), & x_{ii}\in M_{i}\left(k\right):\left|M_{i}\left(k\right)\right|>1,\;\left(10\text{b}\right)\\ \max_{j\in N_{i}}\left(u_{j}\left(k\right)\right)-\Delta T, & x_{ii}\notin M_{i}\left(k\right),\;\left(10\text{c}\right) \end{array} \end{array}$$

where *T*_*leader*_ is the group leader temperature, |*M*_*i*_(*k*)| is the cardinality of the set *M*_*i*_(*k*), $\max_{j\in N_{i}}\left(u_{j}\left(k\right)\right)$ defines the highest temperature setpoint among neighbours of agent *i*, and Δ*T* is a thermal gradient constant. In the case where agent *i* own density value has a unique maximum in its density vector, it becomes the leader Eq (10a). But if the agent’s own density value does not have a unique maximum then the new reference value is equal to the old one Eq (10b). When the agent’s density value is different from the maximum value of its density vector, then its temperature setpoint is equal to the difference between the highest temperature setpoint of its neighbours and the constant value Δ*T* Eq (10c).

### Experimental procedure

The experimental study is carried out in accordance with all relevant regulations, where ethical approval is not required for non-invasive behavioural experiments with invertebrates. One experimental run takes 30 minutes, which is enough time for the honeybees to make a collective decision. The experiments are conducted in two group sizes, namely with 50 or 100 honeybees. Room temperature is maintained in the range between 26-28°C. To record the video of the experiment, we used infra-red diodes for lighting, invisible to honeybees, and an infra-red camera.

**Fig 5:**
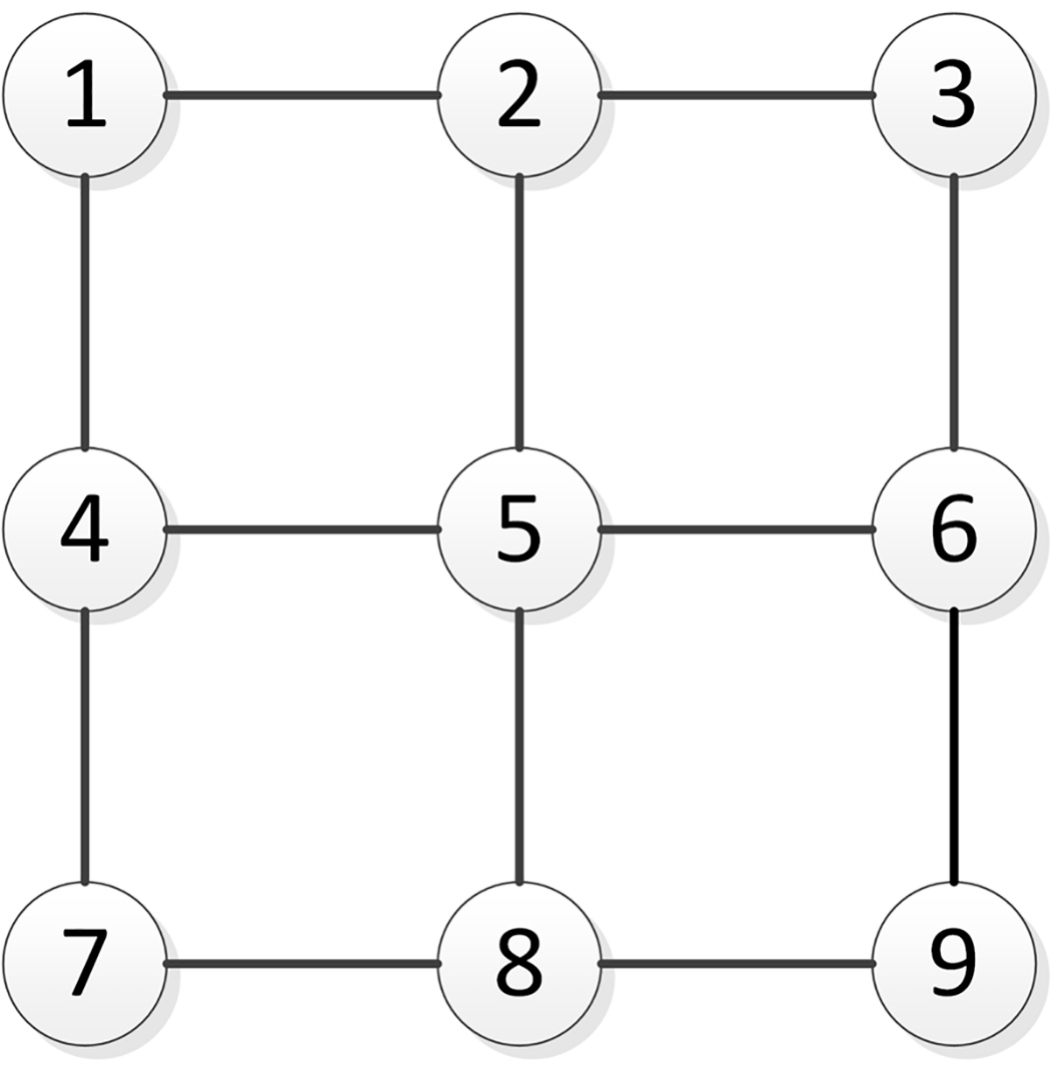
Network topology. The CASUs are spatially distributed according to a two-dimensional grid, where the number denotes CASU’s ID and lines represent communication links.

We consider a network of 9 CASUs organized in rectangular pattern, with a distance between neighbouring CASUs of 90 mm, and enclosed within a circular wall with a diameter of 350 mm. The network topology, where circles represent the CASUs and lines represent created communication links between them, is given by Fig 5.

To ensure that the environmental conditions do not influence the collective behaviour, the experiments have been performed in the dark, and the floor, where honeybees can move, has been covered with wax sheets [26, 31]. The wax is replaced before the start of each experiment to remove any odor or hormone traces, should there be any leftover from the previous run. The honeybees are randomly placed in the arena at the beginning of each experiment with initial temperature of all CASUs set to 26°*C*. The gradient controller constant is experimentally determined and is set to Δ*T* = 6°*C*, where the minimum temperature reference value of the CASUs is equal to 26°*C*.

Bidirectional interaction between the developed multi-robot system and the honeybee society creates a mixed group of two structurally different collective adaptive systems. Distributed operation principles of both systems lead to a collective decision making of the mixed society, which has been experimentally tested in two experiment types: (I) without, and (II) with CASU failure in the multi-robot system. In the experiment with failure, we simulated a failure of a CASU upon honeybee aggregation around it. The objective of this experiment is to validate adaptation ability of the mixed group to the failure of a group member. The mixed group should find a new CASU where honeybees will aggregate.

## Results

### Experiment I: Cooperative behaviour of the mixed society

**Fig 6:**
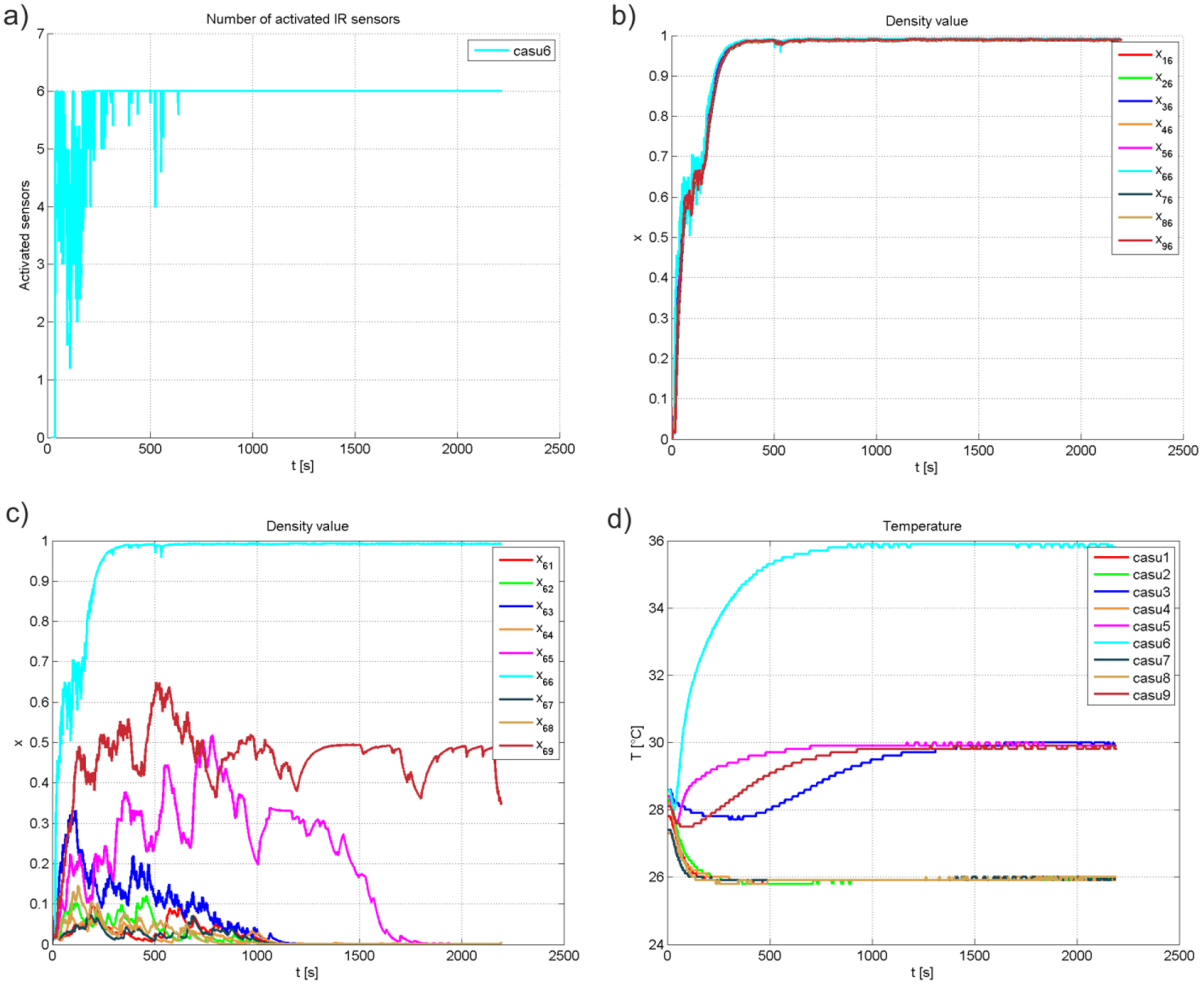
Responses of the conducted experiments. (a) The number of CASU 6 activated IR sensors. (b) Density values of CASU 6 as seen by other CASUs. (c) Density values of all CASUs as seen by CASU 6. (d) Temperature responses.

In this experiment, cooperative abilities of the developed interactive multi-robot system have been validated with a group of 100 honeybees. According to the number of activated IR sensors of CASU 6 presented in Fig 6a, it dominates over other agents in the network. Fig 6b shows responses of density value of CASUs in the network with regard to CASU 6. It can been seen that the density value of each CASU follows the measured density value of CASU 6 (*x*_66_). Thus, graph connectivity guarantees convergence of the density value of each CASU to a value measured by the CASU which is updating its density value, as stated in Eq (5a). Based on density vector of CASU 6 with regard to others (Fig 6c), the gradient controller determines CASU 6 as the temperature leader (Fig 6d). Other CASUs calculate their temperature setpoints knowing the highest temperature of their neighbours according to the Eq (10). CASU 3, 5 and 9 are neighbours of the leading CASU and their temperature setpoint after the transient period is equal to 30°*C*.

**Fig 7:**
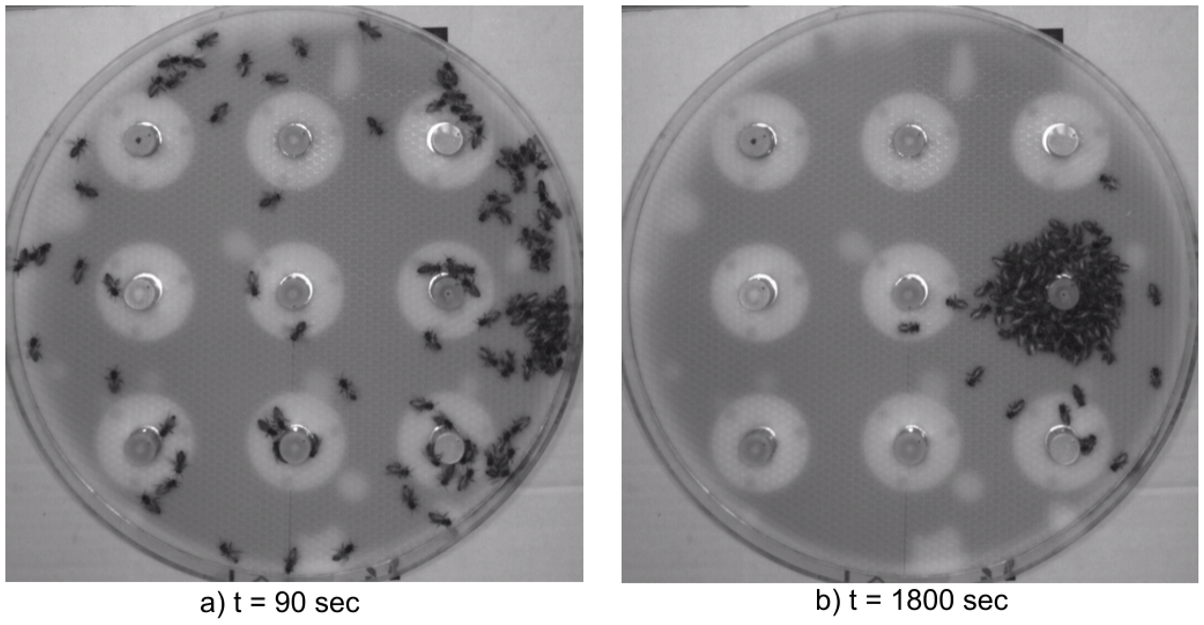
Different snapshots of experimental validation. (a) At t = 90 s the honeybees are randomly moving inside the arena. (b) At t = 1800 s the honeybees have aggregated around CASU 6.

Fig 7 presents snapshots taken in different moments of the experiment. At the beginning, the honeybees are moving across the arena triggering IR sensors of the CASUs. The experiment ends with a chosen temperature leader in the multi-robot system and the aggregation of the majority of honeybees around the leading CASU.

### Experiment II: Cooperative and adaptive behaviour of the mixed society

**Fig 8:**
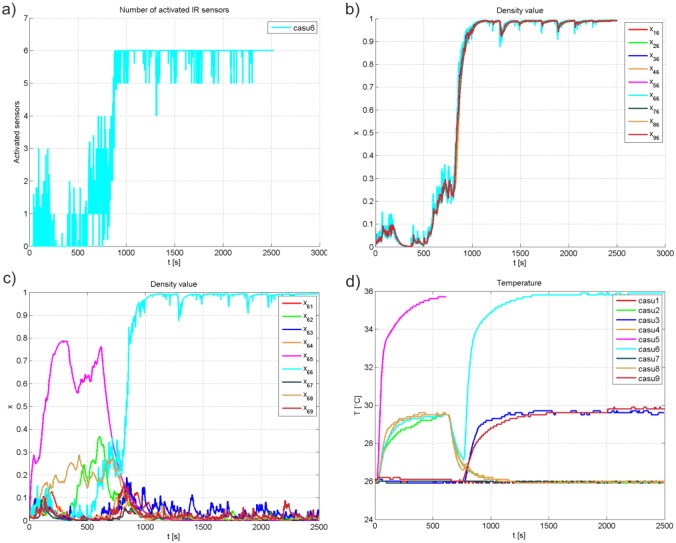
Responses of the conducted experiment with CASU 5 failure. (a) The number of CASU 6 activated IR sensors. (b) Density values of CASU 6 as seen by other CASUs. (c) Density values of all CASUs as seen by CASU 6. (d) Temperature responses.

**Fig 9:**
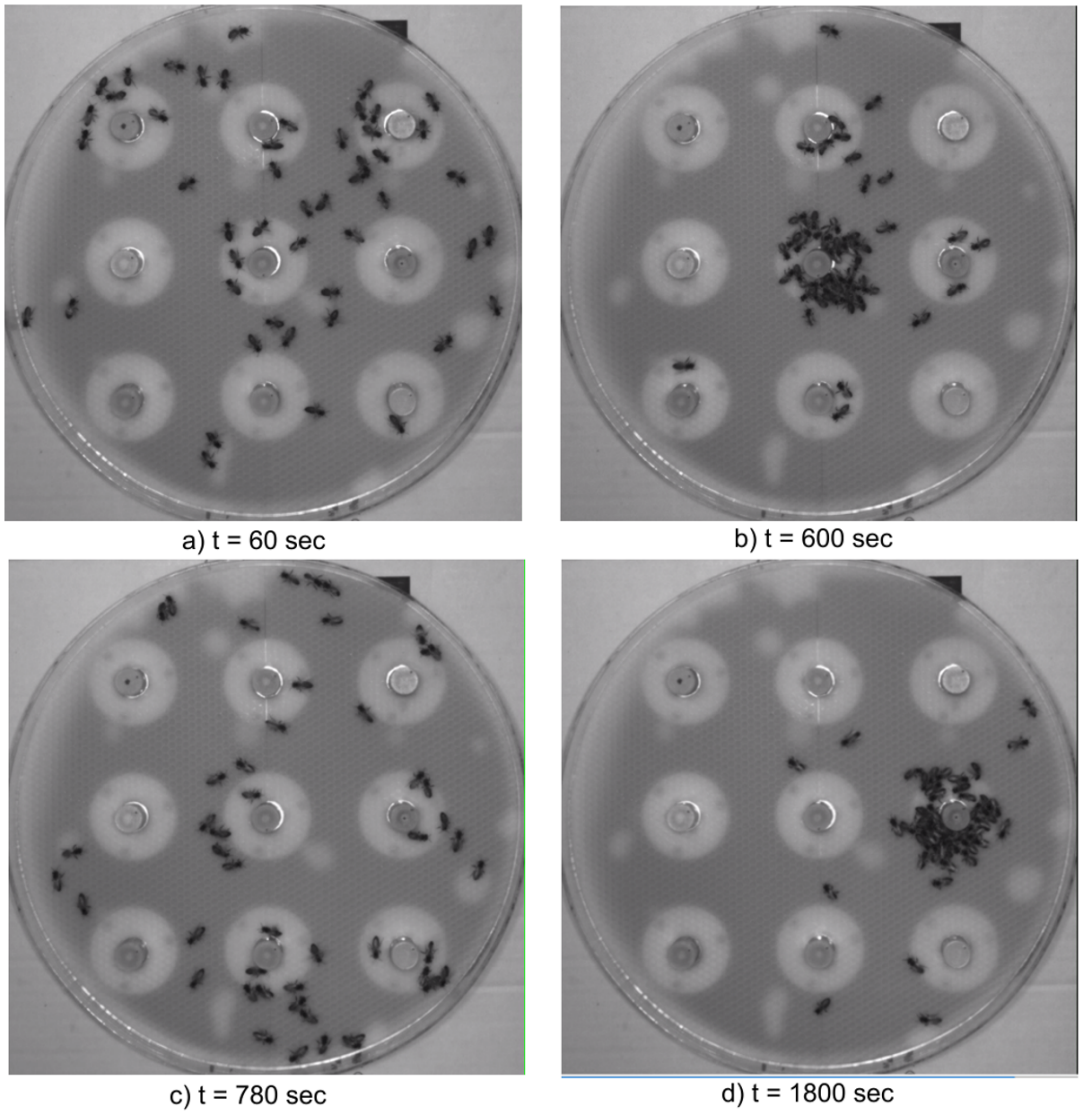
Different snapshots of experimental validation. (a) At t = 90 s the honeybees are randomly moving inside the arena. (b) Honeybees have aggregated around CASU5. (c) Honeybees are searching for a new CASU. (d) CASU6 becomes the new aggregation point.

In this experiment, collective decision of a mixed society has been reached with a group of 50 honeybees and simulated failure of a certain CASU. The network of CASUs and honeybees must adapt to external disturbance and find a new leader in the network. Fig 8 presents responses of relevant system’s variables, including the number of activated sensors of CASU 6 (a), density value of CASU 6 as seen by other CASUs in the network (b), density value of CASU 6 regarding other CASUs in the network (c), and temperature response of each CASU (d). Fig 9 shows snapshots taken in key moments of the experiments. At the beginning of the experiment, the honeybees are randomly distributed inside the arena. Since the density value of CASU 5 is the highest among others, as presented in Fig 8a, it becomes the leader and gathers the majority of honeybees in its vicinity. After its domination, the failure of the CASU 5 has been provoked at *t* = 600 sec, and its temperature setpoint response is not presented after the failure. Its temperature decreases to the ambient temperature resulting in disintegration of the aggregated honeybee group and individuals start to move inside the arena, as seen in Fig 9c. In the absence of communication with CASU 5, its density value seen by other CASUs in the network approaches zero. Moving honeybees trigger presence detection of CASUs, where in the conducted experiment presence density measurement of CASU 6 dominates and it becomes the new leader. Density values of CASUs in the network with regard to CASU 6 shows convergence of density values to the value of CASU 6. Fig 9d shows the new aggregation around CASU 6 at the end of the experiment.

## Discussion

A cooperation of the developed multi-robot system and social insects on a common objective is achieved through formation of a thermal gradient in the network of CASUs under the control of collective decisions made by honeybee groups. The experimental setup is such that the CASUs are unaware of the total number and distribution of honeybees in the arena, but only operate based on the local information of density of the honeybees based on the data acquired by proximity sensors. However, the local density measurement of an individual CASUs is propagated through the network in a decentralized manner using a consensus algorithm. The thermal gradient is then formed by CASUs, again in a decentralized manner, by setting the temperature setpoints of individual CASUs based on the obtained local density values.

The experiments validating the cooperation of two fundamentally different collective systems, differing in structure, operation principles, size and properties were conducted using a network of 9 CASUs and varying number of honeybees. The size of the honeybee group does not seem to affect collective decision significantly. Additionally, the experiments demonstrate the adaptive functionality of the proposed decentralized controller. Namely, the network of robots shown capable of adaptation and formation of a new collective decision in the case of robot failure.

We demonstrated that with suitable mechatronic design of a network of static combined actuators sensors units, guided by different types of physical stimuli and decentralized properties of multi-robot systems, interaction between the artificial units and honeybees can be achieved. The powerful control techniques used to coordinate the actions of the developed multi-robot system bring novel insights into methods for extracting principles of collective behaviour in social insects.

In the future, we will use technological advances of the designed multi-robot system that can adapt its actions autonomously based on the measured states in animal behaviour to investigate the reaction of honeybees to implemented stimulations. Understanding these interactive sequences that can manipulate the emergence of complex behaviours in social animals offer a new way to model and evaluate collective behaviour in animal societies. We expect to gain new insights by conducting experiments that explore interactive, cooperative and coordination abilities of the designed full scale arena. Since information exchange and control algorithms are essential factors in successful coordination and cooperation in multi-robot systems, we will implement control strategies that ensure fulfillment of the system’s objective. Finally, we expect that the knowledge extracted from the work in this field can enhance the distributed system control in real world applications on a large scale, with the hope of bringing the prospects such as autonomous traffic or nano-robotics closer to realization and everyday application.

## Supporting information

S1 Technical Documentation Supporting material covers technical requirements, mechanical and electronic design of the developed experimental setup and CASU. (PDF)
