# Supplementary material for: A robotic system for researching social integration in honeybees
Source: PLoS One. 2017 Aug 9;12(8):e0181977. doi: 10.1371/journal.pone.0181977 (PMC5549902; doi:10.1371/journal.pone.0181977)
Supplement: S1 Technical Documentation — (PDF) [file pone.0181977.s001.pdf]

A robotic system for researching social integration in honeybees
Supporting information

Karlo Griparić[1], Tomislav Haus[1], Damjan Miklić[1], Marsela Polić[1], Stjepan Bogdan[1]

1 University of Zagreb, Faculty of Electrical Engineering and Computing, Zagreb, Croatia

1 Experimental setup design

An artificial unit that would be capable of interaction with honeybees should be able to both produce different physical stimuli, and measure relevant environmental values. Based on the available data and experimental observations of the collective behaviour of honeybees, three types of stimuli are chosen that can influence attractive, stopping and repellant behaviour of honeybees, implemented using heat, vibration, and airflow. Technical requirements of actuators and sensors used for generating a certain type of stimulation are listed in Table A.

Table A. List of actuators and sensors.

**Actuator/Sensor type**	**Required range**	**Accuracy**
Heat	$\theta \in [25, 45]^\circ C$	$\pm 0.25^\circ C$
Vibrations	$f \in [0, 1000]$Hz	±10Hz
Airflow	$p_{in} \in [3, 4]$ bar	Adjustable output flow
Presence detection	$d \in [1, 20]$ mm	±0.5 mm

Taking into account the small size of the honeybee and interactive requirements for the robot unit, it becomes obvious that the robot design is very challenging due to the fact that a large number of electronic components must be packed into a small volume. To cover the area of the proposed experimental setup, a network of cooperating CASUs has been developed, presented in Fig. A. The setup contains an array of static actuator-sensor units situated inside a circular arena where interaction with honeybees can emerge.

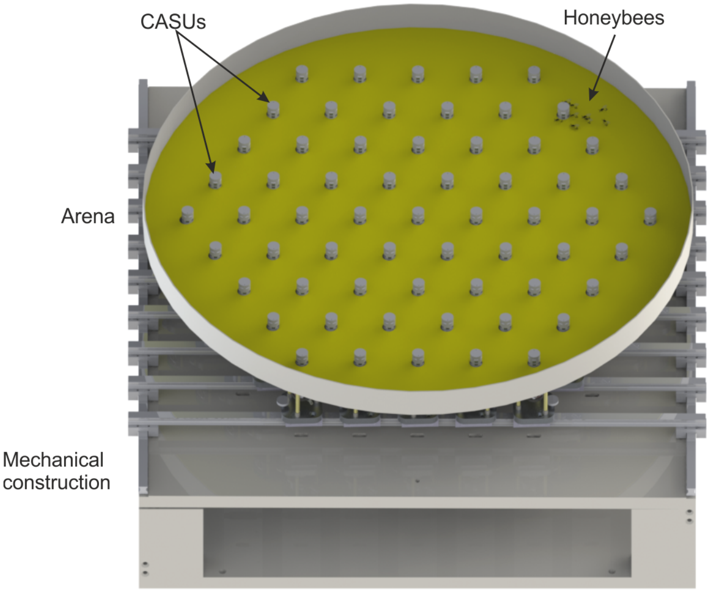

Fig A. An overview of the experimental setup.

1.1 Arena for interaction with honeybees

The mechanical construction of the setup consists of two square plastic plates, whose edge length is 900 mm, mounted one above the other. The vertical distance of 300 mm between the plates, created using four legs separating the plates on their vertices, leaves enough space to place the necessary components between them.

The upper side of the top plate is equipped with the railing mechanism with a grid of nine horizontally and two vertically adjustable linear rails. The carriage on which each CASU is mounted can then be readily moved along the M-shaped rail line. A single direction of carriage movement, along the rail length, is ensured with bearing elements between the rail and the carriage. The carriages can however be locked in certain positions along the rail, by applying a vertical force on the rail surface using a screw mounted on the carriage. Movement and placement of CASUs along the rails are constrained by CASU and grid dimensions, resulting in minimum 65 mm distance between two carriages, while the maximal distance depends on the number of CASUs placed on the same rail. The M-shape of the rail side section restricts addition and removal of carriages to the end of the rail.

The bottom side of the top plate houses single board control computers, the *Beaglebones*, in order to keep them physically close to the CASUs due to power consuption and communication requirements. On the other hand, since the

computers dissipate a significant amount of heat, this positioning at the same time provides thermal isolation of the experimental arena, taking advantage of the low thermal conductivity of plastic.

There are several common components for all of the CASUs in the network, including main power supply, local Ethernet network infrastructure, air valves with control board, and workstation. The bottom side of the top plate is additionally milled to form parallel grooves for cable and air pipes routing for communication with common components, and to provide housing for air flow regulators.

1.2 Mechanical design

The developed CASU with component descriptions and dimensions is presented in Fig. B. An aluminium ring, 60 mm wide, separates the designed robotic unit into visible and non-visible part.

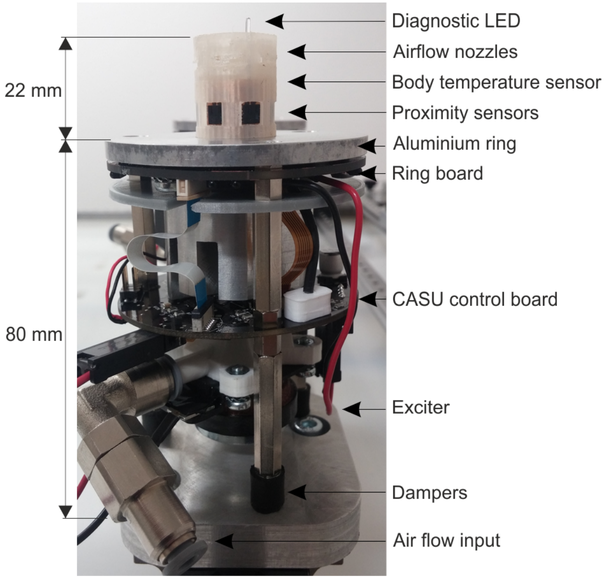

Fig B. The developed CASU. Parts descriptions and dimensions.

The visible part, sized comparable to the honeybee size, is designed in a form of plastic circular body, with a diameter of 18.5 mm. It contains a diagnostic light, six proximity sensors, a temperature sensor, and nozzles for airflow. The diagnostic light source, i.e. an RGB LED, is actually mounted on the control board, because of the limited space and high heat dissipation, and the light is conducted to the top of the CASU with an optical fiber cable. Infrared proximity sensor, with integrated measuring procedure based on built-in transmitter and receiver on a single chip, has been selected. Its maximal detection distance does not exceed 20 mm, but cannot be uniquely determined due to uncertainties in

infrared beam reflection on different environmental obstacles. To guarantee the coverage of the complete area around the body, six sensors have been soldered on a flexible PCB and folded inside the plastic housing with holes where the sensors are positioned. The body temperature sensor on the top of the CASU is used for ambient temperature measurement, being the only temperature sensor in the design that does not measure temperature of a physical part of the CASU.

The non-visible part houses the CASU actuator components, along with computation units and other accessory components. The aluminium ring on the top separating the visible and non-visible parts of the CASU, serves both for conduction of heat, and as a housing for the ring board, which contains temperature sensors and accelerometer. The ring temperature is controlled in closed loop by Peltier module, with feedback provided from temperature sensors mounted by the upper side of the module. The Peltier module is situated directly below the aluminium ring. It produces a temperature difference on its surfaces when direct current is applied to it, heating one side of the module while the other side becomes cooler. Its heat flux direction can be controlled by changing the direction of the current through the module. During the action of cooling, the heat produced by the noncontrolled side of the Peltier (i.e. the lower side, without feedback loop) is conducted away through an aluminium heatsink below the ring, assisted by an electric fan mounted on the heatsink side, which generates an air stream.

The non-visible part of the CASU also contains actuator parts responsible for vibration and airflow stimuli. The honeybee freezing effect can be triggered using an exciter, realized in a form of electromagnetic coil with an oscillatory mass mounted in such a way that its mass oscillates in the direction perpendicular to the arena floor. Vibrations can be represented as a periodic exchange of potential and kinetic energy of a mechanical system. In the case of forced vibration, an external periodic force is applied to the system, given by the equation:

$$F(t) = F_0 sin(\omega t + \delta_0), \tag{1}$$

where F_0 is the amplitude of vibration, ω is the angular frequency, t is the time, and δ_0 is the initial phase angle. The system reaches the oscillation frequency equal to the frequency of the applied force after a transient response. This results in periodic vertical displacement of the arena floor above the aluminium ring, and can be used for local freezing effect stimulation. In order to prevent vibration spreading across the arena setup, as well as consequential coupled vibrations in the arena, rubber dampers have been installed between the carriers and the arena.

The repelling stimulus for the bees is implemented as an air stream coming out from the nozzles situated on the top of CASUs body. A plastic pipe directs the air inside the CASU from air system entry point to the nozzles. The air steam is generated by a compressed air system, which is comprised of a central compressor, electro-magnetic valves, flow regulators, pipes, and nozzles. Air flow can be turned on and off by switching the state of the electromagnetic valve between open and closed. Airflow intensity can be tuned manually by an additional valve.

Two control boards are also placed within the non-visible part of the CASU: the already mentioned ring board, screwed to the aluminium ring, and the main control board, placed below the heatsink for thermal isolation.

1.3 Electronic design

Hierarchically designed electronic architecture, divided into two levels, provides a compromised solution between peripheral capabilities for connecting sensors and actuators, and processing power for control program execution. The low-level architecture is based on two microcontrollers with integrated peripheral interfaces for actuator-sensors management and communication with higher level computer. The high-level side of the electronic architecture, based on a *Beaglebone* computer, provides an interface between the high-level software and the low-level microcontroller functionalities through the developed Python API (Application Programming Interface).

The low-level architecture is based on two microcontrollers, and includes a set of printed circuit boards mounted on the CASU construction and connected according to the diagram given in Fig. C. The main microcontroller is based on a dsPIC33 device, and controls all low-level electronic components connected with digital pins or standard buses for chip-to-chip communication. The auxiliary microcontroller, also based on a dsPIC33 device, generates sine signal for driving the exciter, with adjustable amplitude and frequency. Since the microcontroller can generate only discrete voltage states, a sine shape signal is obtained through a pulse width modulation (PWM) interface, with a continuously changing duty cycle in the period of the required signal, and additional filtering of the produced PWM signal with an RC filter.

Aside from the two microcontrollers, the low-level architecture contains current amplifiers for driving RGB LED and heatsink fan, MOSFET transistors connected into two H bridges for driving the Peltier module and the exciter, and a heatsink temperature sensor. The output voltage for LED and fan can be adjusted with two separate PWM channels of the main microcontroller and peripheral drivers that provide sufficient current output. The H bridge circuit consists of four MOSFET transistors, two P-channel and two N-channel transistors, supporting a maximum output current of 3A, chosen based on the required heat stimulation range, as listed in Table A. The heatsink temperature sensor is used for detecting insufficient heat dissipation. If the temperature rises above the limit value, the microcontroller is triggered and turns the Peltier module off.

Measured physical values include the aluminium ring temperature, amplitude and frequency of mechanical vibrations and presence detection of honeybees in the vicinity of the CASU. The ring's temperature is obtained by averaging temperatures measured by four digital sensors. Multiple sensors provide increased accuracy and redundancy. Vibration amplitude and frequency are calculated using FFT (Fast Fourier Transformation) from acceleration forces acquired by digital accelerometer. Infrared distance sensors are connected to the main microcontroller through an I^2C bus multiplexor, which resolves the problem of

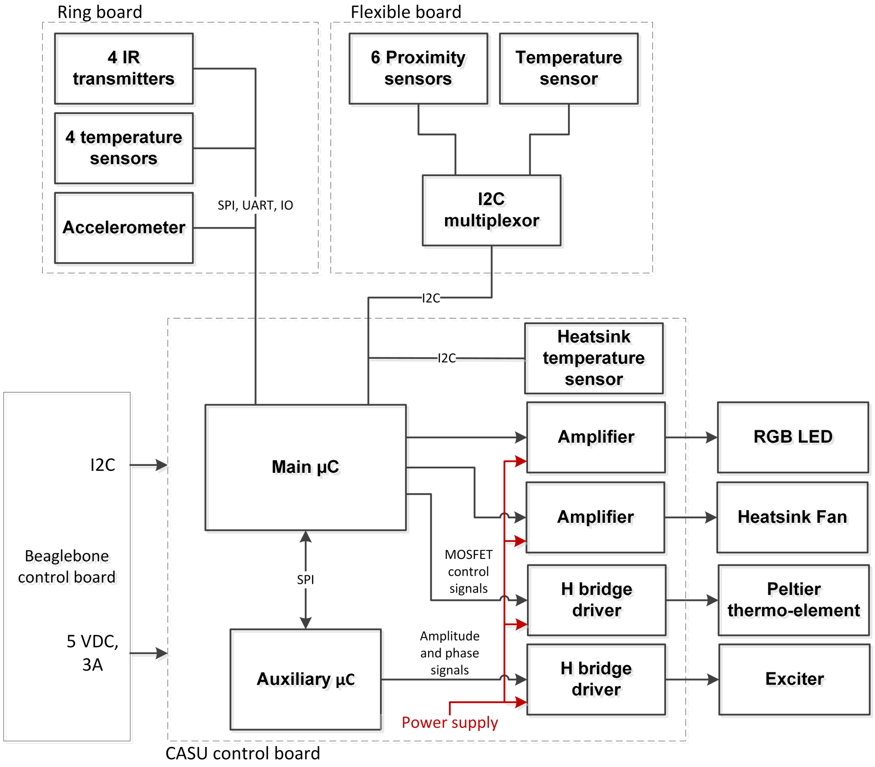

Fig C. Low-level electronic architecture. CASU's low-level architecture connects and controls all electronic components mounted on three PCB boards: CASU control, ring and flexible boards.

connecting several devices with the same address on the same bus.

The high-level architecture is based on a *Beaglebone* computer, and serves as an interface between the high-level software and the low-level microcontroller. The computer independently executes user programs and communicates with other CASU user programs on the network, and interfaces to the remote work-station, where programming and monitoring functionalities are provided.

In order to guarantee information exchange between neighbouring robots, beside Ethernet network, an additional communication channel, integrated on the low-level side, is implemented using infrared communication module. It supports asynchronous serial communication, at maximum speed of 115 kb/s, without the need for fixed infrastructure and node addressing. The position of the module, situated under the level of the arena floor and directed towards nearest neighbour, ensures the quality of communication without influence of external disturbances including reflection of infrared light from proximity sensors or physical barrier.
